# Analysis of fluid force and flow fields during gliding in swimming using smoothed particle hydrodynamics method

**DOI:** 10.3389/fbioe.2024.1355617

**Published:** 2024-05-23

**Authors:** Meng-Meng Liu, Chuan-Wen Yu, Qing-Hua Meng, Xiao-Fan Hao, Zhi-Long Chen, Ming He

**Affiliations:** ^1^ Department of Physical Education, Dongshin University, Naju, Republic of Korea; ^2^ School of Physical Education and Health, Heze University, Heze, China; ^3^ Tianjin Key Laboratory of Sports Physiology and Sports Medicine, Tianjin University of Sport, Tianjin, China; ^4^ Tianjin Key Laboratory of Port and Ocean Engineering, Tianjin University, Tianjin, China

**Keywords:** swimming, gliding, fluid force, flow field, smoothed particle hydrodynamics (SPH), numerical analysis

## Abstract

Gliding is a crucial phase in swimming, yet the understanding of fluid force and flow fields during gliding remains incomplete. This study analyzes gliding through Computational Fluid Dynamics simulations. Specifically, a numerical model based on the Smoothed Particle Hydrodynamics (SPH) method for flow-object interactions is established. Fluid motion is governed by continuity, Navier-Stokes, state, and displacement equations. Modified dynamic boundary particles are used to implement solid boundaries, and steady and uniform flows are generated with inflow and outflow conditions. The reliability of the SPH model is validated by replicating a documented laboratory experiment on a circular cylinder advancing steadily beneath a free surface. Reasonable agreement is observed between the numerical and experimental drag force and lift force. After the validation, the SPH model is employed to analyze the passive drag, vertical force, and pitching moment acting on a streamlined gliding 2D swimmer model as well as the surrounding velocity and vorticity fields, spanning gliding velocities from 1 m/s to 2.5 m/s, submergence depths from 0.2 m to 1 m, and attack angles from −10° to 10°. The results indicate that with the increasing gliding velocity, passive drag and pitching moment increase whereas vertical force decreases. The wake flow and free surface demonstrate signs of instability. Conversely, as the submergence depth increases, there is a decrease in passive drag and pitching moment, accompanied by an increase in vertical force. The undulation of the free surface and its interference in flow fields diminish. With the increase in the attack angle, passive drag and vertical force decrease whereas pitching moment increases, along with the alteration in wake direction and the increasing complexity of the free surface. These outcomes offer valuable insights into gliding dynamics, furnishing swimmers with a scientific basis for selecting appropriate submergence depth and attack angle.

## 1 Introduction

Gliding in swimming refers to the forward movement of a swimmer, without using arms or legs for propulsion but achieved solely through inertia. To minimize resistance, a streamlined posture characterized by outstretched arms, overlapping hands, straightened legs, closed feet, and flexed plantar is typically adopted (Lyttle et al., 1998; Naemi et al., 2010). Gliding occurs during the start, between strokes, and after turns. It accounts for 10%–25% of the pool length or the distance travelled in a race (Chatard et al., 1990; Morais et al., 2019), thus significantly affecting the swimmer’s performance.

Existing studies on gliding mainly focus on the drag force acting on the swimmer. Since this drag force excludes the active drag (Ungerechts and Niklas, 1994; Zamparo et al., 2009) produced by the swimmer’s propulsion, it is commonly referred to as passive drag. Furthermore, passive drag can be separated into three components: form (or pressure) drag, frictional (or viscous) drag, and wave drag (Lyttle et al., 2000; Ungerechts and Arellano, 2011). There are generally three methodologies employed in the investigation of passive drag, namely, towing trial, flume test, and velocity decay method (Scurati et al., 2019).

Through towing trials (i.e., gliding with constant velocities in still water), Lyttle et al. (1998) examined the impact of gliding velocity and submergence depth on passive drag. Zamparo et al. (2009) investigated the role of trunk inclination and projected frontal area in passive drag, noting that these factors are closely related to the gliding velocity. By conducting flume tests (i.e., stationary in steady and uniform flows), Vennell et al. (2006) quantified the contribution of wave drag to passive drag under various submergence depths. Chatard and Wilson (2008) reported that wearing either a full-body suit or a waist-to-ankle suit reduced passive drag, thereby enhancing the swimmer’s performance. Zaïdi et al. (2008) analyzed the effects of the swimmer’s head position on velocity profiles, hydrodynamic drag, and streamline patterns. Marinho et al. (2009) compared passive drag between two gliding postures: arms extended at the front and arms alongside the trunk. Using the velocity decay method (i.e., gliding with decreasing velocities in still water), Kjendlie and Stallman (2008) showed that adults experienced greater passive drag than children due to their larger body sizes. Barbosa et al. (2015) evaluated the relative contributions of form drag and frictional drag to total passive drag. Li et al. (2017a) studied the hydrodynamic characteristics and surrounding vortex structures of a swimmer gliding with six degrees of freedom. For more information, the reader is referred to the review papers of Naemi et al. (2010) and Scurati et al. (2019).

Although numerous studies have been conducted on passive drag during gliding, there is a lack of research on other fluid force components, especially under various gliding velocities, submergence depths, and attack angles. In fact, vertical force determines the difficulty of maintaining a desired submergence depth, while pitching moment affects the stability of gliding. Understanding their characteristics under different conditions holds profound importance in guiding gliding. Moreover, despite a streamlined posture, complex turbulent flow still exists around the swimmer. While most existing studies focus on the flow fields around flexing limbs (Ungerechts et al., 2000; Arellano et al., 2002; Matsuuchi et al., 2009; Pacholak et al., 2014; Shimojo et al., 2019; Yang et al., 2021; Tanaka et al., 2022), minimal attention has been paid to those around the swimmer during gliding. Additionally, experimental studies, despite remaining the primary research approach to date, possess inherent limitations. These include high costs associated with facilities and swimmers, challenges in controlling conditions like attack angle and submergence depth, and constraints in acquiring data, particularly in flow visualization. With the advancement of Computational Fluid Dynamics (CFD), numerical analysis of gliding is progressively emerging as a trend (Silva et al., 2008; Zaïdi et al., 2010; Marinho et al., 2011; Costa et al., 2015; Zhan et al., 2015; Wang and Kabala, 2022).

This study aims to analyze the passive drag, vertical force, and pitching moment acting on the swimmer during streamlined gliding as well as the surrounding velocity and vorticity fields using a CFD method named Smoothed Particle Hydrodynamics (SPH). The SPH method is a fully Lagrangian meshless technique that discretizes the continuum domain into a finite number of particles and calculates the field variations through interactions among neighboring particles. Originally introduced for astrophysics (Gingold and Monaghan, 1977; Lucy, 1977), it has since been extended to a wide range of fields such as hydrodynamics (Huang et al., 2022), geophysics (Onyelowe et al., 2023), biophysics (Zhang et al., 2021), electromagnetics (Ala et al., 2006), elastic and plastic dynamics (Greto and Kulasegaram, 2020), and explosion mechanics (Ming et al., 2016).

Regarding swimming, Cohen et al. (2012) simulated dolphin kick swimming using the SPH method and explored the effects of ankle flexibility and kick frequency on propulsion and flow structures. Cohen et al. (2015) investigated the associations of thrust with hand trajectories, orientations, and velocities during freestyle stroke. They also found that the vortices generated by hands and transferred towards legs enhanced propulsion. Cohen et al. (2018) identified the moment when peak arm thrust occurs and examined the impact of stroke frequency on the thrust contributions of arms and legs. Recently, Cohen et al. (2020) analyzed the effects of body kinematic asymmetry caused by unilateral breathing on the fluid force and velocity of a freestyle swimmer. These precedents demonstrate the adaptability and reliability of the SPH method in swimming research.

## 2 Methods

### 2.1 Swimmer model

**FIGURE 1:**
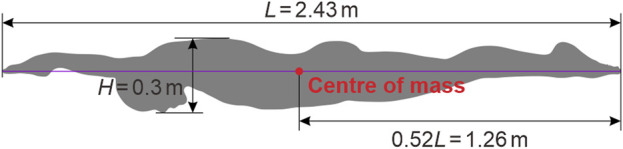
Simplified 2D swimmer model with a streamlined prone posture.

A simplified 2D version of the 3D swimmer model introduced by Li et al. (2017a), Li et al. (2017b) is employed as depicted in Figure 1. The 3D swimmer model was constructed based on the mean anthropometrical characteristics of a group of Chinese male swimmers, featuring a streamlined prone posture with outstretched arms, overlapping hands, straightened legs, closed feet, and flexed plantar. Standing at a height of 1.82 m, the 3D swimmer model possessed a finger-to-toe length (*L*) of 2.43 m, with upper and lower extremity lengths of 0.8 m and 0.91 m respectively, shoulder and pelvis breadths of 0.42 m and 0.34 m respectively, and cheat, waist, hip, thigh, and crus circumferences of 0.98 m, 0.79 m, 0.92 m, 0.58 m, and 0.38 m respectively. Additionally, the frontal projected height (*H*) was 0.3 m, the surface area was 1.93 m^2^, and the volume was 0.08 m^3^. The mass was 81.87 kg, with the centre of mass positioned at a distance of 0.52*L* from the toe, and the moments of inertia for roll, pitch, and yaw of 0.90 kg⋅m^2^, 19.61 kg⋅m^2^, and 20.01 kg⋅m^2^ respectively.

### 2.2 SPH model

This section introduces the SPH model for flow-object interaction, encompassing its governing equations, boundary conditions, and time integrator. It is worth noting that real gliding refers to the movement of a swimmer in still water. However, to reduce the computational domain and thereby enhance computational efficiency, a steady and uniform flow is generated in a free-surface channel. The swimmer model is immobilized in the flow direction while vertically translating with a constant velocity in the case of an attack angle.

#### 2.2.1 Governing equations

For weakly compressible and barotropic fluids, the governing equations consist of the continuity, Navier-Stokes, state, and displacement equations, which can be written as Eqs 1–4:

$$\frac{D\rho }{Dt}=-\rho \nabla ∙u$$ (1)

$$\frac{Du}{Dt}=-\frac{\nabla p}{\rho }+g+\nu \nabla^{2}u$$ (2)

$$p=c_{0}^{2}\left(\rho -\rho_{0}\right)$$ (3)

$$\frac{Dr}{Dt}=u$$ (4)

where *t* is the time; *ρ*, *p*, and *ν* are the density, pressure, and kinematic viscosity, respectively; ***u*** and ***r*** are the velocity and position, respectively; ***g*** is the gravitational acceleration; *ρ*_0_ is the reference density taken to be 1,000 kg/m^3^; *c*_0_ is the numerical speed of sound (Sun et al., 2019) determined by Eq. 5:

$$c_{0}=10\max\left(U_{\max},\sqrt{p_{\max}/\rho_{0}}\right)$$ (5)

with *U*_max_ and *p*_max_ being the maximum velocity and pressure, respectively. In the present study, *U*_max_ is roughly set as the gliding velocity, and *p*_max_ approximately equals the hydrostatic pressure at the channel bottom.

In the SPH framework, the discrete forms of Eqs 1–4 (Antuono et al., 2012) are Eqs 6–9:

$$\frac{D\rho_{i}}{Dt}=-\rho_{i}\sum_{j}\left(u_{j}-u_{i}\right)∙\nabla_{i}W_{ij}V_{j}+\delta hc_{0}\sum_{j}\psi_{ij}∙\nabla_{i}W_{ij}V_{j}$$ (6)

$$\frac{Du_{i}}{Dt}=-\frac{1}{\rho_{i}}\sum_{j}\left(p_{j}+p_{i}\right)\nabla_{i}W_{ij}V_{j}+g+\alpha hc_{0}\frac{\rho_{0}}{\rho_{i}}\sum_{j}\pi_{ij}\nabla_{i}W_{ij}V_{j}$$ (7)

$$p_{i}=c_{0}^{2}\left(\rho_{i}-\rho_{0}\right)$$ (8)

$$\frac{Dr_{i}}{Dt}=u_{i}$$ (9)

where subscripts *i* and *j* refer to a pair of interacting particles; *V* is the particle volume; *W*_*ij*_ = *W* (***r***_*i*_ − ***r***_*j*_, *h*) is the Wendland C2 kernel function (Wendland, 1995) with *h* being the smoothing length.

The last term of Eq. 6 performs a density diffusive role, helping eliminate numerical noise. *δ* is a tuned coefficient usually taken to be 0.1. ***ψ***_*ij*_ is calculated by Eq. 10:

$$\psi_{ij}=2\left[\left(\rho_{j}-\rho_{i}\right)-\frac{1}{2}\left({\langle \nabla \rho \rangle }_{j}^{L}+{\langle \nabla \rho \rangle }_{i}^{L}\right)∙\left(r_{j}-r_{i}\right)\right]\cdot \frac{r_{j}-r_{i}}{{\left|r_{j}-r_{i}\right|}^{2}}$$ (10)

where ${\langle \nabla \rho \rangle }^{L}$ denotes the renormalized density gradient (Randles and Libersky, 1996) defined as Eq. 11:

$${\langle \nabla \rho \rangle }_{i}^{L}=\sum_{j}\left(\rho_{j}-\rho_{i}\right)B_{i}\cdot \nabla_{i}W_{ij}V_{j}$$ (11)

with ***B**_i_* being calculated by Eq. 12:

$$B_{i}={\left[\sum_{j}\left(r_{j}-r_{i}\right)⊗\nabla_{i}W_{ij}V_{j}\right]}^{-1}$$ (12)

The last term of Eq. 7 provides shear and bulk viscosities, helping to stabilize the numerical scheme and reduce spurious oscillations. *α* = 8*ν*/(*hc*_0_) is adopted to reproduce the shear viscosity of a real fluid (Monaghan, 2005). *π*_*ij*_ is given by Eq. 13:

$$\pi_{ij}=\frac{\left(u_{j}-u_{i}\right)∙\left(r_{j}-r_{i}\right)}{{\left|r_{j}-r_{i}\right|}^{2}}$$ (13)

For gliding in swimming, vortices are generated behind the swimmer model. Vortex-induced low pressure can trigger tensile instability and even result in numerical cavitation (Sun et al., 2018). To address this issue, the optimized particle shifting scheme proposed by Khayyer et al. (2017) is adopted. Specifically, after each time step, fluid particles are shifted from regions of high concentration to regions of low concentration. The displacement vector is calculated by Eq. 14:

$$\delta r_{i}=\left\{\begin{array}{ll} -C_{s}h^{2}\nabla_{i}C_{i}\; & i\in \text{inner particles }\\ -C_{s}h^{2}\left(I-{\tilde{n}}_{i}⊗{\tilde{n}}_{i}\right)∙\nabla_{i}C_{i} & i\in \text{free}‐\text{surface particles and free}‐\text{surface vicinity particles}\\ 0\; & i\in \text{splash particles } \end{array}\right.$$ (14)

where *C*_*s*_ is a shifting coefficient taken to be 0.5; ***I*** is the identity matrix; $\nabla_{i}C_{i}$ is the gradient of the particle concentration defined as Eq. 15:

$$\nabla_{i}C_{i}=\sum_{j}V_{j}\nabla_{i}W_{ij}$$ (15)

${\tilde{n}}_{i}$ is a corrected unit normal vector calculated by Eq. 16:

$${\tilde{n}}_{i}=-\frac{B_{i}∙\nabla C_{i}}{\left|B_{i}∙\nabla_{i}C_{i}\right|}$$ (16)

The upper limit of the shifting distance is set as 0.2*h* (Lind et al., 2012).

Free-surface particles are detected based on the position vector divergence criterion, i.e., $\nabla_{i}∙r_{i}<1.5$ . Free-surface vicinity particles are identified if ${1.5\leq \nabla }_{i}∙r_{i}<2$ and $\left|r_{i}-r_{j}\right|\leq h$ , where subscripts *i* and *j* denote the free-surface vicinity particle and its nearest free-surface particle, respectively. Splash particles are flagged if $\nabla_{i}∙r_{i}<1.5$ and $\left|r_{i}-r_{j}\right|>2h$ , where subscripts *i* and *j* denote the splash particle and free-surface vicinity particle, respectively. Any particles that are not categorized as free-surface, free-surface vicinity, or splash particles are inner particles.

#### 2.2.2 Free-surface boundary

Two conditions need to be met at the free surface: kinematic and dynamic conditions. The kinematic condition stipulates that, in the direction normal to the free surface, the velocity of the free-surface particle is equal to the rate of change in the free-surface position. This can be implicitly verified due to the Lagrangian nature of the SPH method (Colagrossi et al., 2009). The dynamic condition requires that the pressure remains constant at the free surface. This is also satisfactory because the weakly compressible SPH method manages to assign zero pressure to the free surface via Eq. 8 (Violeau and Rogers, 2016).

#### 2.2.3 Solid boundary

**FIGURE 2:**
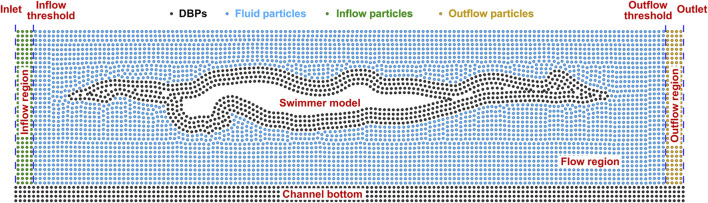
Illustration of the boundary conditions of the SPH model.

Solid boundaries are implemented using the modified dynamic boundary particles (DBPs) suggested by Ren et al. (2015). As illustrated in Figure 2, four rows of DBPs are positioned at the channel bottom and along the contour of the swimmer model. The separation between adjacent rows and that between adjacent DBPs in the same row are set as the initial particle spacing. All DBPs participate in Eqs 6, 8 as fluid particles. The calculated density is further smoothed by the mean density of fluid particles within the kernel support (Cheng et al., 2021) as Eq. 17:

$${\tilde{\rho }}_{k}=\chi \rho_{k}+\left(1-\chi \right)\frac{1}{N_{p}}\sum_{i=1}^{N_{p}}\left[\rho_{i}+\frac{\rho_{0}g}{c_{0}^{2}}∙{\left(r_{i}-r_{k}\right)}_{z}\right]$$ (17)

where subscripts *k* and *i* refer to a DBP and its neighbouring fluid particle, respectively; subscript *z* denotes the vertical component; ${\tilde{\rho }}_{k}$ is the corrected density; *N*_*p*_ is the total number of fluid particles within the kernel support; *χ* is a weighted coefficient varying from 0 to 0.5 (He et al., 2021; He et al., 2023).

DBPs at the channel bottom are not included in Eq. 7, so they remain stationary over time. However, DBPs along the contour of the swimmer model are used to calculate the fluid force acted on the DBP as Eq. 18:

$$f_{k}=m_{k}\frac{Du_{k}}{Dt}=-\sum_{i}\left(p_{i}+{\tilde{p}}_{k}\right)\nabla_{k}W_{ki}V_{k}V_{i}+\alpha hc_{0}\rho_{0}\sum_{i}\pi_{ki}\nabla_{k}W_{ki}V_{k}V_{i}$$ (18)

where ${\tilde{p}}_{k}$ is the corrected pressure of the DBP based on Eqs 8, 17. By summing up ***f***_*k*_ of each DBP along the contour, the fluid force acting on the swimmer model can be obtained by Eq. 19:

$$F=\left(D_{P},F_{L}+F_{B}\right)=\sum_{k}f_{k}$$ (19)

where ***D***_***P***_, ***F***_***L***_, and ***F***_***B***_ are the passive drag, lift force, and buoyancy, respectively. Correspondingly, the pitching moment is calculated by Eq. 20:

$$M_{P}=\sum_{k}\left(r_{k}-r_{m}\right){\times f}_{k}$$ (20)

where ***r***_*m*_ is the position of the center of mass. In contrast to the DBPs at the channel bottom, those along the contour of the swimmer model move synchronously with the swimmer model.

#### 2.2.4 Inflow and outflow boundaries

A steady uniform flow is generated using the inflow and outflow boundaries described by Federico et al. (2012). As illustrated in Figure 2, inflow and outflow regions, both with a length of 2*h*, are situated at the upstream and downstream ends of the free-surface channel, respectively. The horizontal velocities of inflow particles are prescribed according to the gliding velocity and the attack angle, while their vertical velocities are zero. Besides, hydrostatic pressure is assigned to inflow particles. Once an inflow particle crosses the inflow threshold, it turns into a fluid particle and takes part in the governing equations presented in Section 2.2.1. A new inflow particle is inserted at the same time. It is located at the same vertical position as the converted particle, and its horizontal distance from the inlet is equal to the horizontal distance between the converted particle and the inflow threshold. Fluid particles that cross the outflow threshold become outflow particles. They have the same velocities as inflow particles, while their density and pressure are frozen. Once an outflow particle crosses the outlet, it is removed from the computational domain.

Inflow and outflow velocities must also be assigned to fluid particles at the beginning of the computation. This ensures the smooth entries of inflow particles into the flow region, preventing any obstructions by fluid particles that could lead to a rise in the free surface near the inflow threshold. This also enables fluid particles to enter the outflow region smoothly, avoiding a discontinuity in particle distribution caused by velocity mismatches between fluid and outflow particles.

#### 2.2.5 Time integrator

A Symplectic integrator with 2nd-order accuracy (Monaghan, 2005) is taken for time stepping. As an explicit scheme, the time step complies with the Courant-Friedrich-Levy condition and a viscosity condition (Monaghan and Kos, 1999) as Eq. 21:

$${∆t}_{c}=0.2\min_{i}\left(\frac{h}{c_{0}+h\max_{j}\pi_{ij}}\right)$$ (21)

It is also dependent on a forcing term (Monaghan, 1992) and a viscous-diffusion condition (Morris et al., 1997), written as Eqs 22, 23:

$${∆t}_{f}=0.2\min_{i}\sqrt{\frac{h}{\left|Du_{i}/Dt\right|}}$$ (22)

$${∆t}_{v}=0.125\frac{h^{2}}{\nu }$$ (23)

Finally, the time step is taken as Eq. 24:

$$\Delta t=\min\left({∆t}_{c},{∆t}_{f},{∆t}_{v}\right)$$ (24)

### 2.3 Laboratory experiment

It is crucial to ensure the reliability of the SPH model prior to simulating gliding in swimming. Since a 2D SPH model is employed, replicating 3D experiments on swimmers’ gliding is not feasible. Instead, a laboratory experiment on a circular cylinder advancing steadily beneath a free surface (Miyata et al., 1990), which can be regarded as a 2D problem, is adopted.

The experiment was carried out in an 86-m-long, 3.5-m-wide, and 2.4-m-deep water tank at the University of Tokyo, Japan. The cylinder was fixed in a 2.4-m-long, 0.5-m-wide, and 0.7-m-deep channel, which was towed at a constant velocity (***U***) of 0.3 m/s within the water tank. The radius of the cylinder (*R*) was 0.08 m. The ratio of the submergence depth (*d*_*s*_) to *R* ranged from 1 to 4.5.

### 2.4 Numerical setups

This section presents the setups of two groups of numerical simulations. The first group is tailored to validate the reliability of the SPH model by replicating the laboratory experiment described in Section 2.3. The second group is designed to analyze fluid force and flow fields during gliding in swimming.

#### 2.4.1 Simulation of moving cylinder

**FIGURE 3:**
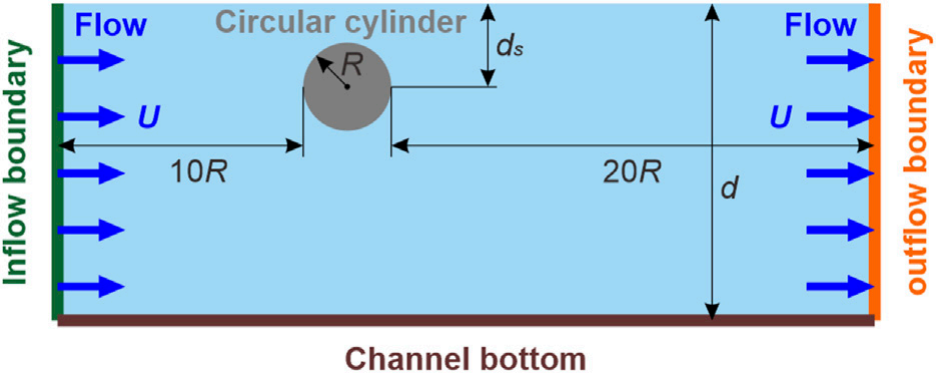
Sketch of the numerical setup of a circular cylinder advancing steadily beneath a free surface.

The moving cylinder was simulated in the free-surface channel depicted in Figure 3. The circular cylinder, with *R* = 0.08 m, was placed 10*R* and 20*R* distances from the inflow and outflow boundaries, respectively. The water depth (*d*) was 0.7 m. The inflow and outflow velocities were set as ***U*** = 0.3 m/s, corresponding to the Reynolds number *Re* = 2| ***U*** |*R*/*ν* = 4.96×10^4^ and the Froude number *Fr* = | ***U*** |/(2 ***g*** *R*)^1/2^ = 0.24. As validation examples, only three representative conditions were considered: *d*_*s*_/*R* = 1.125, 1.5, and 3. Initial particle spacing (*δ*_*p*_) was chosen as 1/60 of the diameter of the cylinder, resulting in a total number of 254 K thousand particles.

***D***_***P***_ and ***F***_***L***_ versus *d*_*s*_/*R* were computed and compared with experimental data in the dimensionless forms of drag coefficient (*C*_*D*1_) and lift coefficient (*C*_*L*_) defined as Eqs 25, 26:

$$C_{D1}=\frac{\bar{D_{P}}}{\rho_{0}\left|U\right|URW_{s}}$$ (25)

$$C_{L}=\frac{\bar{F_{L}}}{\rho_{0}\left|U\right|URW_{s}}$$ (26)

where $\bar{D_{P}}$ and $\bar{F_{L}}$ are the low-pass filtered ***D***_***P***_ and ***F***_***L***_, respectively, with positive directions pointing towards the outflow boundary and the channel bottom; *W*_*s*_ is the spanwise width of the cylinder.

#### 2.4.2 Simulation of gliding in swimming

**FIGURE 4:**
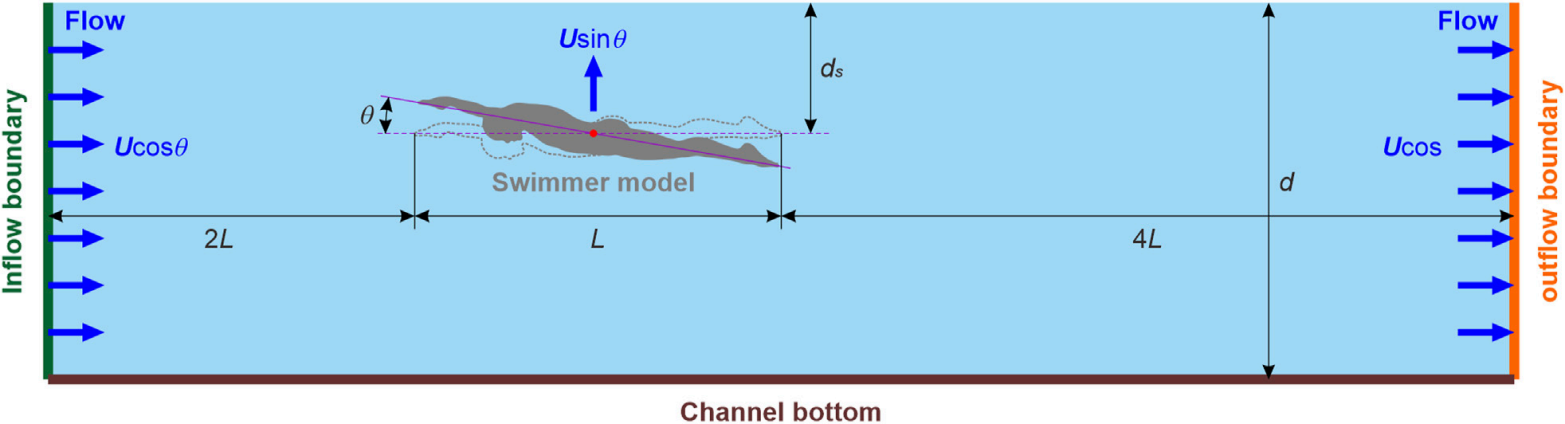
Sketch of the numerical setup of gliding in swimming.

Gliding in swimming was simulated in the free-surface channel illustrated in Figure 4. The swimmer model, with *L* = 2.43 m and attack angles (*θ*) = −10°, −5°, 0, 5°, and 10°, was positioned 2*L* and 4*L* distances from the inflow and outflow boundaries, respectively. *θ* is defined as the angle between the flow direction and the line connecting the finger to the toe, with a positive value indicating a pitch-up posture (i.e., upper limb up and lower limb down). The absence of large upstream and downstream spaces is justified by the short duration of gliding, as the flow field perturbations by gliding have not yet reached the inflow and outflow boundaries. *d* was 2 m, adhering to the FINA facilities rules of swimming facilities for Olympic Games and World Championships. To account for the various influences of wave drag, *d*_*s*_ ranged from 0.2 m to 1.0 m with an interval of 0.2 m. The gliding velocity ***U***) varied between 1 m/s and 2.5 m/s in an increment of 0.5 m/s. This covers the velocities of swimmers during the start, between strokes, and after turns (Tor et al., 2015; Barbosa et al., 2018), and corresponds to *Re* = | ***U*** |*L*/*ν* = 2.43×10^6^–6.08×10^6^ and *Fr* = | ***U*** |/(***g*** *L*)^1/2^ = 0.205–0.512. As an equivalent, the inflow and outflow velocities were set as ***U*** cos*θ*. The swimmer model was immobilized in the flow direction while vertically translating with a constant velocity of ***U*** sin*θ*. *δ*_*p*_ was chosen as *H*/60 of the swimmer model, i.e., 0.005 m, leading to a total number of 1.346 million particles.

***D***_***P***_, ***F***_***L***_ + ***B***, and ***M***_***P***_ under various ***U***, *d*_*s*_, and *θ* were computed and analyzed in the low-pass filtered forms of $\bar{D_{P}}$ , $\bar{F_{L}+B}$ , and $\bar{M_{P}}$ as well as in the dimensionless forms of drag coefficient (*C*_*D*2_), vertical coefficient (*C*_*V*_), and pitching coefficients (*C*_*P*_) defined as Eqs 27–29:

$$C_{D2}=\frac{\bar{D_{P}}}{0.5\rho_{0}\left|U\right|UL}$$ (27)

$$C_{V}=\frac{\bar{F_{L}+B}}{0.5\rho_{0}\left|U\right|UL}$$ (28)

$$C_{P}=\frac{\bar{M_{P}}}{0.5\rho_{0}\left|U\right|UL^{2}}$$ (29)

where, positive directions of $\bar{F_{L}+B}$ and $\bar{M_{P}}$ point towards the free surface and aligning with *θ*, respectively. Additionally, velocity and vorticity fields surrounding the swimming model were visualized, where the vorticity a fluid particle is defined as the curl of the velocity field (Monaghan, 1992) given by Eq. 30:

$$\omega_{i}={\left(\nabla \times u\right)}_{i}=\sum_{j}\frac{m_{j}}{\rho_{i}}\left(u_{i}-u_{j}\right)\times \nabla_{i}W_{ij}$$ (30)

Thus, a positive ***ω*** denotes anticlockwise rotation and a negative ***ω*** signifies clockwise rotation.

## 3 Results

### 3.1 Reliability of SPH model

**FIGURE 5:**
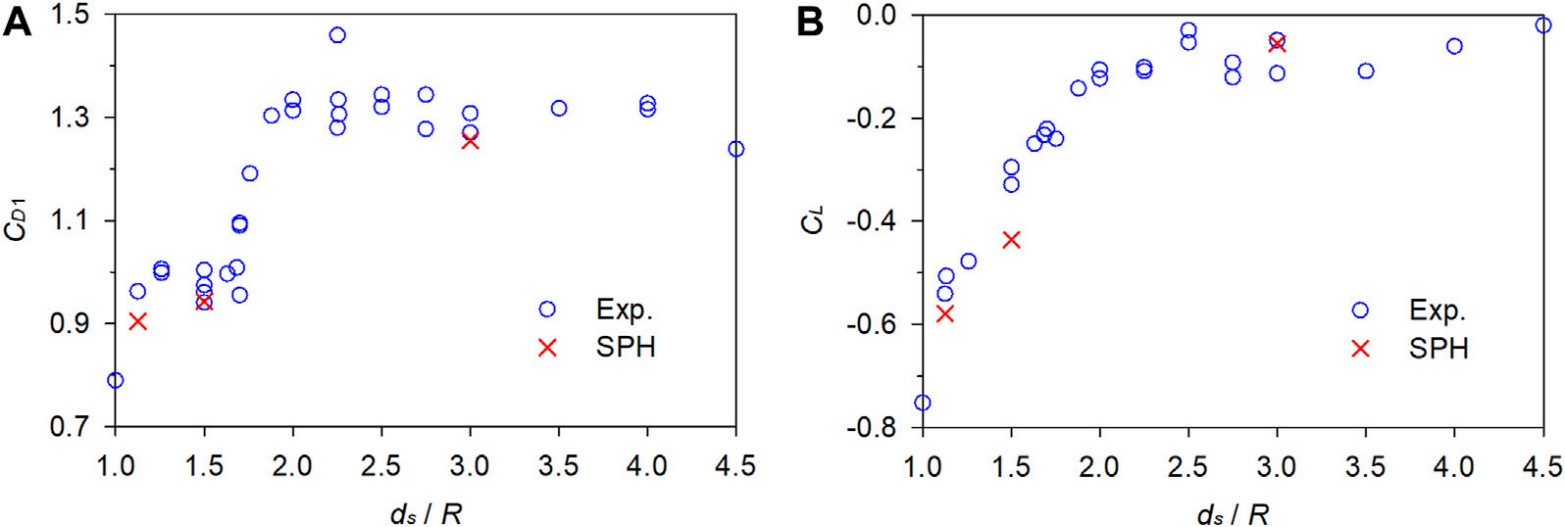
Comparison between the numerical and experimental fluid force coefficients. **(A)** Drag coefficient. **(B)** Lift coefficient.

Figure 5 presents a comparison between the numerical *C*_*D*1_ and *C*_*L*_ and the experimental data obtain by Miyata et al. (1990). Although the computations were conducted solely for three specific conditions, namely, *d*_*s*_/*R* = 1.125, 1.5, and 3, the numerical *C*_*D*1_ and *C*_*L*_ correctly capture the trends of how the experimental data vary with respect to *d*_*s*_/*R*. Furthermore, the numerical values are close to the experimental data, indicating the reliability of the established SPH model for flow-object interaction.

### 3.2 Effects of gliding velocity

**FIGURE 6:**
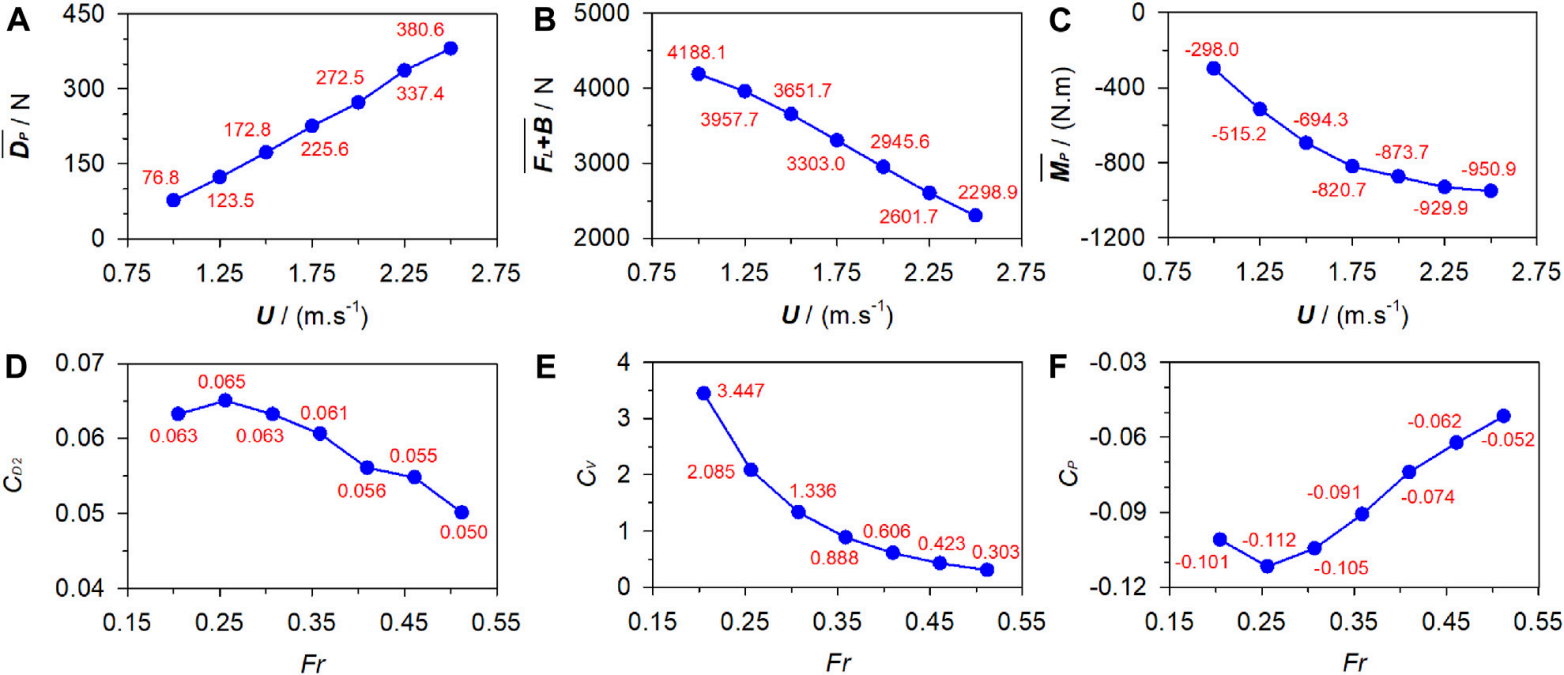
Fluid force and corresponding coefficients under various gliding velocities. **(A)** Passive drag. **(B)** Vertical force. **(C)** Pitching moment. **(D)** Drag coefficient. **(E)** Vertical coefficient. **(F)** Pitching coefficient.

With *d*_*s*_ and *θ* fixed at 0.6 m and 0, respectively, Figure 6 plots the numerical $\bar{D_{P}}$ , $\bar{F_{L}+B}$ , and $\bar{M_{P}}$ under ***U*** = 1 m/s ∼ 2.5 m/s as well as *C*_*D*2_, *C*_*V*_, and *C*_*P*_ under corresponding *Fr* = 0.205–0.512. $\bar{D_{P}}$ increases with the increasing ***U***, while *C*_*D2*_ generally exhibits an opposite trend. Both $\bar{F_{L}+B}$ and *C*_*V*_ decrease as ***U*** increases, but the decreasing trend of *C*_*V*_ tends to flatten out compared to $\bar{F_{L}+B}$ . $\bar{M_{P}}$ is negative and its magnitude increases with ***U***. Conversely, the absolute value of *C*_*P*_ decreases with the increasing ***U*** overall.

**FIGURE 7:**
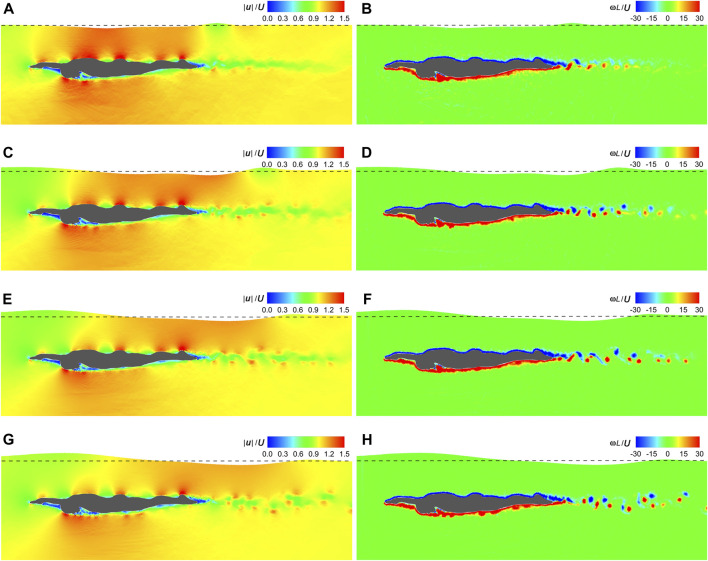
Velocity and vorticity fields under various gliding velocities. **(A)** Velocity field under ***U*** = 1.0 m/s. **(B)** Vorticity field under ***U*** = 1.0 m/s. **(C)** Velocity field under ***U*** = 1.5 m/s. **(D)** Vorticity field under ***U*** = 1.5 m/s. **(E)** Velocity field under ***U*** = 2.0 m/s. **(F)** Vorticity field under ***U*** = 2.0 m/s. **(G)** Velocity field under ***U*** = 2.5 m/s. **(H)** Vorticity field under ***U*** = 2.5 m/s.

Figure 7 shows the velocity and vorticity fields at the dimensionless time instant (*t*| ***U*** |/*L*) = 1 under ***U*** = 1 m/s ∼ 2.5 m/s. High and low velocities occur on the protruding parts and in the sheltered regions of the swimmer, respectively. In contrast, the dorsal side is covered with negative ***ω*** and the ventral side is dominated by positive ***ω***. There is a low-velocity region behind the swimmer, where positive and negative vortices alternate. The further away from the swimmer, the weaker the vortex intensity and the larger the spacing between vortex centers. With the increasing ***U***, the vortex intensity grows stronger and the low-velocity region becomes discontinuous. Additionally, the free surface above the upper torso rises while the surface downstream of the swimmer lowers.

### 3.3 Effects of submergence depth

**FIGURE 8:**
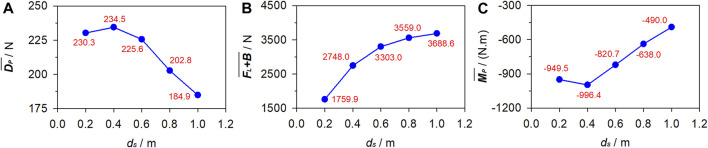
Fluid force under various submergence depths. **(A)** Passive drag. **(B)** Vertical force. **(C)** Pitching moment.

With ***U*** and *θ* fixed at 1.75 m/s and 0, respectively, Figure 8 depicts the numerical $\bar{D_{P}}$ , $\bar{F_{L}+B}$ , and $\bar{M_{P}}$ under *d*_*s*_ = 0.2 m–1.0 m. $\bar{D_{P}}$ generally decreases with the increasing *d*_*s*_. As *d*_*s*_ increases, $\bar{F_{L}+B}$ exhibits a growing trend, although this growth tends to flatten out. $\bar{M_{P}}$ is negative and its magnitude decrease with the increase in *d*_*s*_ overall.

**FIGURE 9:**
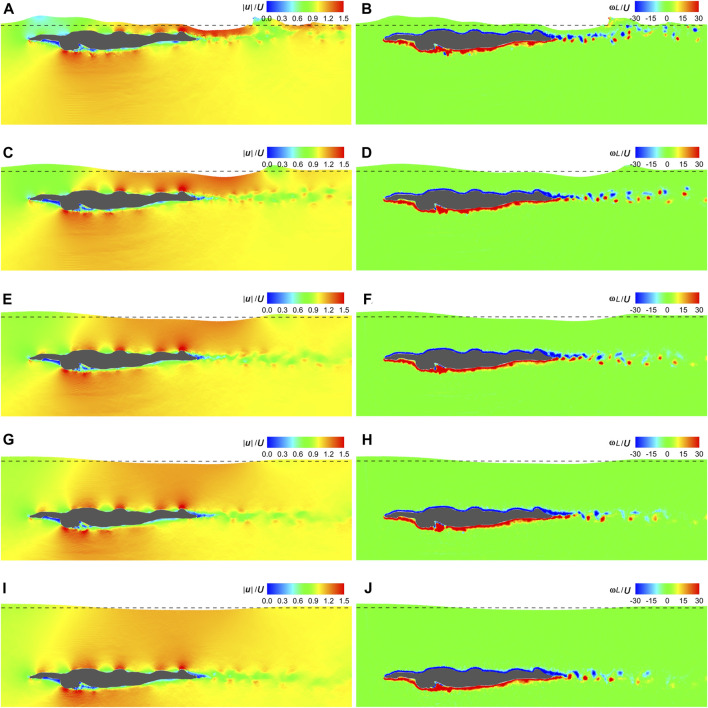
Velocity and vorticity fields under various submergence depths. **(A)** Velocity field under *d*_*s*_ = 0.2 m. **(B)** Vorticity field under *d*_*s*_ = 0.2 m. **(C)** Velocity field under *d*_*s*_ = 0.4 m. **(D)** Vorticity field under *d*_*s*_ = 0.4 m. **(E)** Velocity field under *d*_*s*_ = 0.6 m. **(F)** Vorticity field under *d*_*s*_ = 0.6 m. **(G)** Velocity field under *d*_*s*_ = 0.8 m. **(H)** Vorticity field under *d*_*s*_ = 0.8 m. **(I)** Velocity field under *d*_*s*_ = 1.0 m. **(J)** Vorticity field under *d*_*s*_ = 1.0 m.

Figure 9 displays the velocity and vorticity fields at *t*| ***U*** |/*L* = 1 under *d*_*s*_ = 0.2 m–1.0 m. Irrespective of *d*_*s*_, the dorsal and ventral sides of the swimmer are coated with negative and positive vorticities, respectively. However, as *d*_*s*_ decreases, the high-velocity regions on the dorsal wrist and upper back as well as the low-velocity region on the toe gradually disappear. Additionally, with the decreasing *d*_*s*_, the free surface becomes more complex. It is closer in shape to the dorsal side of the swimmer and breaks downstream. The breaking free surface disturbs the tailing vortices behind the swimmer, resulting in irregularities in their positions, sizes, and shapes.

### 3.4 Effects of attack angle

**FIGURE 10:**
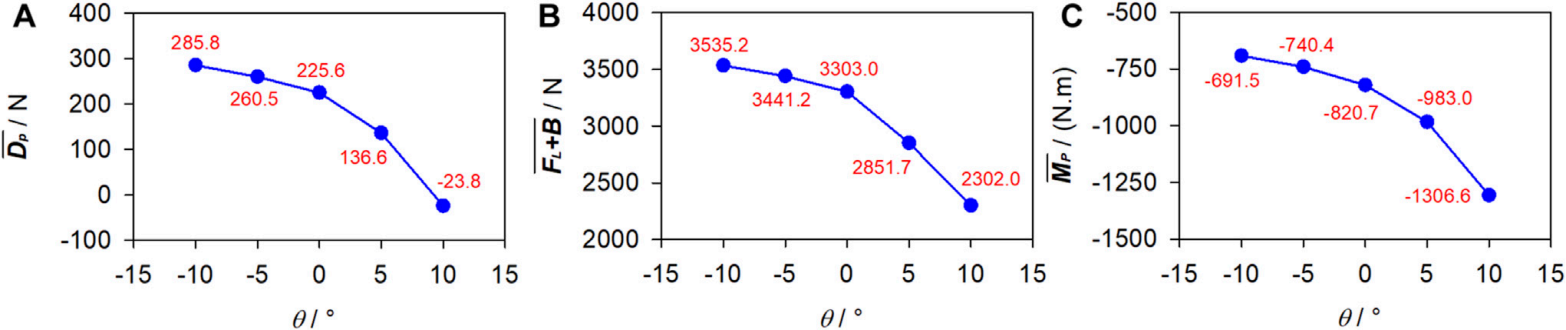
Fluid force under various attack angles. **(A)** Passive drag. **(B)** Vertical force. **(C)** Pitching moment.

With ***U*** and *d*_*s*_ fixed at 1.75 m/s and 0.6 m, respectively, Figure 10 presents the numerical $\bar{D_{P}}$ , $\bar{F_{L}+B}$ , and $\bar{M_{P}}$ under *θ* = −10°–10°. $\bar{D_{P}}$ decreases with the increasing *θ* (mathematically, a positive *θ* is larger than a negative one) and even becomes negative at *θ* = 10°. $\bar{F_{L}+B}$ decreases rapidly as *θ* increases. $\bar{M_{P}}$ is negative and its magnitude exhibits an accelerated growth with the increasing *θ*.

**FIGURE 11:**
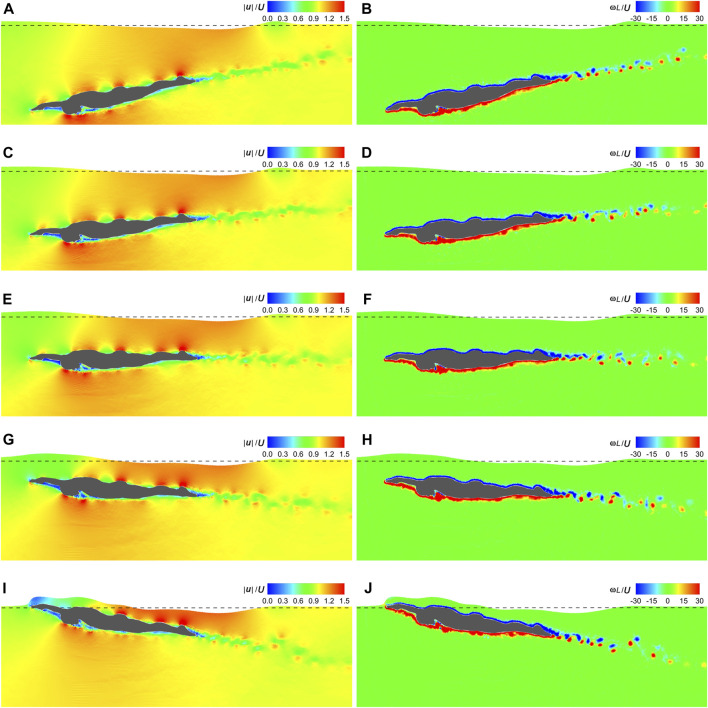
Velocity and vorticity fields under various attack angles. **(A)** Velocity field under *θ* = −10°. **(B)** Vorticity field under *θ* = −10°. **(C)** Velocity field under *θ* = −5°. **(D)** Vorticity field under *θ* = −5°. **(E)** Velocity field under *θ* = 0. **(F)** Vorticity field under *θ* = 0. **(G)** Velocity field under *θ* = 5°. **(H)** Vorticity field under *θ* = 5°. **(I)** Velocity field under *θ* = 10°. **(J)** Vorticity field under *θ* = 10°.

Figure 11 visualizes the velocity and vorticity fields at *t*| ***U*** |/*L* = 1 under *θ* = −10°–10°. Except for when the swimmer surfaces from water, the surrounding velocity and vorticity distributions are almost unchanged with various *θ*. The low-velocity region and trailing vortex trajectory behind the swimmer are also invariant but align with the same *θ* as that of the swimmer. The free surface maintains a consistent shape for *θ* ≤ 0. However, for *θ* > 0, as the swimmer emerges from the water, the free surface initially resembles its dorsal side and then gradually slides off.

## 4 Discussion

This study aims to investigate $\bar{D_{P}}$ , $\bar{F_{L}+B}$ , and $\bar{M_{P}}$ experienced by the swimmer during streamlined gliding, as well as the examination of the surrounding velocity and vorticity fields, utilizing the SPH method. Earlier studies have extensively studied $\bar{D_{P}}$ (Lyttle et al., 1998; Vennell et al., 2006; Zaïdi et al., 2008; Marinho et al., 2009; Zamparo et al., 2009; Barbosa et al., 2015), but $\bar{F_{L}+B}$ and $\bar{M_{P}}$ have received limited attention. While earlier studies have primarily focused on flow fields around flexing limbs (Ungerechts et al., 2000; Arellano et al., 2002; Matsuuchi et al., 2009; Pacholak et al., 2014; Shimojo et al., 2019; Yang et al., 2021; Tanaka et al., 2022), little attention has been paid to those surrounding the streamlined gliding. Although physical experiments have historically been the predominant approach in earlier studies (Chatard et al., 1990; Benjanuvatra et al., 2002; Mollendorf et al., 2004; Kjendlie and Stallman, 2008; Cortesi et al., 2014; Tor et al., 2015), numerical simulations are increasingly emerging as the preferred method for future research.

$\bar{D_{P}}$ , the most significant fluid force influencing gliding, can be minimized through decreasing ***U*** and increasing *d*_*s*_ and *θ*, as evident from Figures 6A, 8A, 10A. Swimmers are unlikely to voluntarily reduce ***U*** to decrease $\bar{D_{P}}$ ; instead, they can focus on optimizing *d*_*s*_ and *θ*. Lyttle et al. (1998) observed that *d*_*s*_ = 0.4 m can effectively decrease wave drag, leading to a reduction in $\bar{D_{P}}$ . Tor et al. (2015) emphasized the necessity of maintaining a minimum *d*_*s*_ of 0.5 m. Vennell et al. (2006) and Novais et al. (2012) recommended *d*_*s*_ = 0.7 m and 0.75 m, respectively. However, Figure 8A demonstrates that $\bar{D_{P}}$ decreases linearly as *d*_*s*_ increases from 0.4 m to 1.0 m, suggesting that *d*_*s*_ exceeding 1.0 m may be beneficial. On the other hand, Bixler et al. (2007) reported that $\bar{D_{P}}$ consistently opposes the direction of gliding and reaches its minimum value at *θ* = 0. However, Figure 10A reveals that a positive *θ* results in lower $\bar{D_{P}}$ compared to a negative *θ*, and a larger positive *θ* can even cause $\bar{D_{P}}$ to act in the same direction as gliding.

$\bar{F_{L}+B}$ determines the difficulty of maintaining a desired *d*_*s*_. Figures 6B, 8B, and 10B indicate that decreasing *d*_*s*_ and increasing ***U*** and *θ* all contribute to minimizing $\bar{F_{L}+B}$ . Nevertheless, ***U*** is constrained by the proficiency level of the swimmer, leaving only the options to optimizing *d*_*s*_ and *θ*. It is noteworthy that decreasing *d*_*s*_ can simultaneously increase $\bar{D_{P}}$ . Given that swimmers prioritize gliding efficiency over maintaining a desired *d*_*s*_, a deeper *d*_*s*_ is overall preferable. Additionally, increasing *θ* not only decreases $\bar{F_{L}+B}$ but also reduces $\bar{D_{P}}$ . Since there is no conflict between the two objectives, adopting a larger positive *θ* is advisable.

$\bar{M_{P}}$ mainly affects the stability of gliding. Figures 6C, 8C, 10C indicate that minimizing the magnitude of $\bar{M_{P}}$ can be achieved by increasing *d*_*s*_ and decreasing ***U*** and *θ*. However, deliberately decreasing ***U*** to achieve this reduction is impractical, narrowing down the options to optimizing *d*_*s*_ and *θ*. Previous discussions have suggested a deeper *d*_*s*_ through balancing gliding efficiency with maintaining a desired *d*_*s*_. Since a deeper *d*_*s*_ also reduces the magnitude of $\bar{M_{P}}$ , it clearly stands out as a favorable choice. Moreover, while increasing *θ* benefits both gliding efficiency and maintaining the desired *d*_*s*_, it paradoxically increases the magnitude of $\bar{M_{P}}$ . Given that gliding efficiency remains a top priority for swimmers, a larger positive *θ* appears to be the most suitable compromise.

As can be seen in Figures 7, 9, 11, high velocities occur on the protruding parts of the swimmer, including the finger, dorsal wrist, forehead, upper back, buttock, calf, and heel, while in sheltered regions, such as those adjacent to the inner forearm, chest, and toe, velocities are low. This can be attributed to the narrower flow area on the protruding parts, resulting in increased velocities when the flow rate remains constant. Conversely, the broader flow area in the sheltered regions leads to decreased velocities. The dorsal side is covered with negative ***ω*** and the ventral side is dominated by positive ***ω***, with clear boundaries at the finger and the toe. This pattern is due to the friction on the body surface, causing clockwise rotation of fluid near the dorsal side and anticlockwise rotation near the ventral side. Additionally, owing to the shielding effect, a low-velocity region aligned with *θ* is observed at the rear of the swimmer. This region is populated with alternating clockwise and anticlockwise vortices shed from the toe.

Although this study has revealed patterns in how the fluid force and surrounding flow fields vary with gliding velocity, submergence depth, and attack angle, the findings are limited to qualitative guidance for swimmer’s training and competition strategies. This is primarily due to the fact that the present SPH simulations were conducted in 2D, whereas real gliding is 3D. 2D simulations can be interpreted as swimmers having identical cross-sections across unit width, with the cross-section chosen based on the maximum body contour. Consequently, the numerical fluid force is overestimated compared to a 3D swimmer. For instance, Novais et al. (2012) conducted 3D simulations and reported $\bar{D_{P}}$ = 96.51 N and 94.21 N at ***U*** = 2.0 m/s, *θ* = 0, and *d*_*s*_ = 0.5 m and 0.75 m, respectively. In contrast, the present 2D simulations for the same ***U*** and *θ* but *d*_*s*_ = 0.6 m yielded $\bar{D_{P}}$ = 272.5 N. Benjanuvatra et al. (2002) experimentally measured an average $\bar{D_{P}}$ of 50.7 N at ***U*** = 1.6 m/s, *d*_*s*_ = 0.4 m, and *θ* = 0. However, the present 2D simulations for the same *d*_*s*_ and *θ* but ***U*** = 1.75 m/s produced $\bar{D_{P}}$ = 234.5 N. Apart from the overestimation of fluid force, the flow fields obtained from 2D simulations are insufficient to fully represent reality. For example, 3D simulations conducted by Zhan et al. (2015) demonstrated the presence of vortex rings around the swimmer, a feature that was unable to be captured in the present 2D simulations. Therefore, it is necessary to conduct 3D simulations in the future to examine the conclusions drawn herein.

Another limitation of this study lies in its limited portrayal of turbulent flow. As mentioned in Section 2.4.1 and Section 2.4.2, the highest *Re* number reached in the present simulations is 6.08×10^6^, resulting in a remarkably thin boundary layer on the body surface. According to Marrone et al. (2013), a minimum of 10 particles is required to discretize the boundary layer, necessitating a highly refined particle resolution. However, computational efficiency constraints dictated that the particle spacing in this study be set to one-sixtieth of the swimmer’s frontal projected height. Although the alternating vortices at the rear of the swimmer were successfully captured in Figures 7, 9, 11, smaller vortices were omitted, ultimately affecting the accuracy of numerical fluid force. The inclusion of turbulence models offers a crucial means to enhance the detail of turbulence portrayal. While several turbulence models have been developed within the SPH framework, including the *k*-*ε* model (Shao, 2006), sub-particle scale (SPS) model (Lo and Shao, 2002), and SPH-∈ model (Monaghan, 2011), none were employed in this study. The reasons are mainly threefold. Firstly, the particle resolution is insufficient for modeling turbulent flow, and the inclusion of a turbulence model would not significantly alter the numerical results (Mayrhofer et al., 2015; Wang and Liu, 2020). Secondly, as 2D simulations were conducted, the complex 3D turbulent flow could not be truly captured even with a turbulence model. Thirdly, due to the inherent numerical dissipation of the SPH method, a turbulence-free SPH simulation already tends to overestimate turbulence kinetic energy (Lowe et al., 2019). In future studies, the multi-resolution scheme (Sun et al., 2018; 2019) could be incorporated to enhance the local computational accuracy, particularly in regions where turbulence effects are expected to be significant.

## 5 Conclusion

The SPH method was utilized to simulate gliding in swimming under various gliding velocities, submergence depths, and attack angles. The aim was to enhance comprehension of the fluid force acting on the swimmer and the surrounding flow fields. Key findings indicate that as the submergence depth and attack angle increase, the passive drag experienced by the swimmer decreases. However, a deeper submergence depth poses a greater challenge in maintaining a consistent depth, while a larger positive attack angle compromises the stability of gliding. Looking ahead, 3D, multi-resolution SPH simulations are intended to be conducted to further refine the understanding of gliding dynamics, ultimately facilitating more effective swimmer’s training and competition strategies.

## Data Availability

The original contributions presented in the study are included in the article/supplementary material, further inquiries can be directed to the corresponding author.
